# Prediction of TF target sites based on atomistic models of protein-DNA complexes

**DOI:** 10.1186/1471-2105-9-436

**Published:** 2008-10-16

**Authors:** Vladimir Espinosa Angarica, Abel González Pérez, Ana T Vasconcelos, Julio Collado-Vides, Bruno Contreras-Moreira

**Affiliations:** 1 Departamento de Bioquímica y Biología Molecular y Celular, Facultad de Ciencias, Universidad de Zaragoza. Pedro Cerbuna 12, 50009 Zaragoza, España; 2 Instituto de Biocomputación y Física de Sistemas Complejos, Universidad de Zaragoza. Corona de Aragón 42 Edificio Cervantes, 50009 Zaragoza, España; 3 Programa de Genómica Computacional, Centro de Ciencias Genómicas, Universidad Nacional Autónoma de México. Av. Universidad s/n., Colonia Chamilpa 62210, Cuernavaca, Morelos, México; 4 Centro Nacional de Bioinformática. Industria y San José, Capitolio Nacional, CP 10200, Habana Vieja, Ciudad de la Habana, Cuba; 5 Laboratório Nacional de Computação Científica. Av. Getulio Vargas 333, Quitandinha, CEP 25651-075, Petrópolis, Rio de Janeiro, Brasil; 6 Laboratory of Computational Biology, Estación Experimental de Aula Dei, Consejo Superior de Investigaciones Científicas, Av. Montañana 1.005. 50059 Zaragoza, España; 7 Fundación ARAID, Paseo María Agustín 36, Zaragoza, España

## Abstract

**Background:**

The specific recognition of genomic *cis*-regulatory elements by transcription factors (TFs) plays an essential role in the regulation of coordinated gene expression. Studying the mechanisms determining binding specificity in protein-DNA interactions is thus an important goal. Most current approaches for modeling TF specific recognition rely on the knowledge of large sets of cognate target sites and consider only the information contained in their primary sequence.

**Results:**

Here we describe a structure-based methodology for predicting sequence motifs starting from the coordinates of a TF-DNA complex. Our algorithm combines information regarding the direct and indirect readout of DNA into an atomistic statistical model, which is used to estimate the interaction potential. We first measure the ability of our method to correctly estimate the binding specificities of eight prokaryotic and eukaryotic TFs that belong to different structural superfamilies. Secondly, the method is applied to two homology models, finding that sampling of interface side-chain rotamers remarkably improves the results. Thirdly, the algorithm is compared with a reference structural method based on contact counts, obtaining comparable predictions for the experimental complexes and more accurate sequence motifs for the homology models.

**Conclusion:**

Our results demonstrate that atomic-detail structural information can be feasibly used to predict TF binding sites. The computational method presented here is universal and might be applied to other systems involving protein-DNA recognition.

## Background

The specific recognition of genomic *cis*-regulatory elements by nucleic acid binding proteins is of critical importance for many vital processes such as DNA replication and repair, _m_RNA translation and transcriptional regulation. Specific TF-DNA interactions, as well as protein-protein contacts established between closely located TFs at promoter regions, are key determinants of the concerted expression of genes in response to external stimuli. The task of determining the relationships among TFs and their targets has been widely addressed experimentally [1,2] and computationally [3,4]. Of great relevance and use in recent years are computational approaches, based on the construction of statistical models that characterize the DNA-binding preferences of TFs, which are in turn used to scan genomic sequences in an effort to identify new putative binding sites [5,6].

The probabilistic models more commonly used in such computational approaches are position weight matrices (PWMs) obtained from multiple alignments of known binding sites. This approach is limited to TFs with a sufficient number of experimentally identified binding sites, for which reliable statistical models may be built. An alternative approach would be to predict DNA operator sites which are compatible with the mode of binding of a given TF [7]. This approximation would depend on two key components: *i*) the knowledge of the protein and DNA residue positions involved in binding at the spatial level and *ii*) a method to evaluate the compatibility of different DNA bases and amino acids to interact [8,9].

The computational analysis of available protein-DNA complexes has resulted in several papers describing the characteristics of the amino acid-base interactions that determine binding specificity [10-12], the different types of readout mechanisms involved in DNA recognition [13,14] and the evolutionary conservation of residues located at contact interfaces [15-17]. Direct readout is associated to recognition through contacts established between atoms from amino acid side-chains and nitrogen bases. Indirect readout, on the other hand, is mediated by the contribution of residues of the proteins and DNA which are not in direct contact and conformational changes undergone by DNA upon protein binding [14,18]. These reports show that both direct and indirect readouts significantly contribute to specific protein-DNA recognition.

With respect to direct readout, it is estimated that about two-thirds of all protein-DNA interactions are van der Waals contacts which do not generally confer sequence specificity, with the exception of the hydrophobic interactions involving the C7 atom of thymine [12]. On the contrary, hydrogen bonds are the major source of specific interaction contacts. Two-thirds of the hydrogen bonds formed between amino acids and bases are bi-dentate or complex interactions providing a great specificity. Non-classical C-H ···O hydrogen mediated links have also been found at protein-DNA contact interfaces [19], albeit their energetic contributions to recognition are not fully understood yet. These interactions are inherently weak individually [20], which means that cumulative effects are indispensable to significantly account for specificity, as occurs with hydrophobic links.

Water mediated bridges, though common in interaction interfaces, are mostly used as gap-fillers [12] and are also engaged in buffering unfavorable electrostatic repulsions between interacting atoms at the interface [21]. Electrostatic interactions, most of which are established between the protein main chain and the sugar/phosphate backbone, do not significantly contribute to specific recognition, although they play important roles in the transition from the unspecific to the specific complex [22].

To date just a few reports have been published aiming at finding putative TF target sequences using structural information. There have been some attempts to apply physical potential functions – i.e., in the form of atomic force fields – to estimate the energy of interaction and the relative contribution of direct and indirect readout mechanisms [18,23,24]. However, these physical models do not appear to significantly outperform simpler statistical methods [25]. Examples of these methods are approaches oriented to extracting structural information from family-wise comparisons, building a statistical model of the interface derived from crystallographic structures and binding sites of proteins that belong to the same family [26,27]. Other procedures infer statistical potentials of interaction at the residue-base level from datasets of known complexes [7,9,28-30], which are used to examine the compatibility between the protein and its putative sites. However, there is some evidence suggesting that the performance of these methods may be limited by the simplicity of their interaction potentials, calculated at the level of C_α _atoms for protein residues. Moreover, the increasing number of high-resolution structures for protein-DNA complexes opens a new door towards a more detailed study of their contact interfaces. Indeed a recent report confirms that atomic details improve the ability of physical potential functions to predict sequence-specific protein-DNA interactions [25].

In this work we constructed position weight matrices that capture the binding specificity of transcription factors, based on information extracted from the Protein Data Bank (PDB). Three atomic preference matrices, for hydrogen bonds, water-mediated hydrogen bonds and hydrophobic interactions, were derived from a non-redundant training set of 210 complexes annotated in the PDB [31]. These matrices were used to make explicit atomistic representations of hydrogen and hydrophobic bonds as well as the contribution of water molecules at interfaces, which gives us the opportunity to score the direct readout. This contribution is combined with empirical estimations of DNA deformation, in order to calculate a potential of binding that includes both direct and indirect readout contributions. We evaluated the performance of our algorithm in a set of 4 bacterial and 4 eukaryotic TFs which have been co-crystallized bound to DNA, and in most cases our results proved to be as good as or better than those obtained with the structure-based cumulative contact method by Morozov and Siggia [32]. In addition, two TF homology models were analyzed in detail and used to predict their DNA binding motif, after sampling side-chain rotamers at their contact interfaces. In this case the results we obtained were significantly better than those returned by the reference method, which indicates that our algorithm could be suitably used to study TFs of unknown structure starting from structural models. We also discuss the strengths and limitations of our approach that might potentially be used for TFs with few or no experimentally characterized binding sites.

## Results

### Protein-DNA interface atomic contacts and interaction preferences

Starting from a non-redundant training set of crystallographic protein-DNA complexes culled from the PDB, we constructed atomic frequency matrices for hydrogen bonds, water-mediated hydrogen bonds and hydrophobic contacts. A close inspection at the information embodied in these frequency matrices (see Table 1 and Additional file 1) reveals some interesting features of protein-DNA interfaces. For instance, as claimed in previous reports, we found that arginine is the major source of hydrogen bonds, with a marked preference to interact with guanine [12]. However, in the present study we divided this preference in pairs of interacting atoms, finding that groups NH1 and NH2 of arginine establish 88% of the hydrogen bonds with atoms N7 and O6 from guanine. Histidine also showed a marked preference towards guanine, with atom NE2 interacting in 17 out of 35 hydrogen bonds with atoms O6 and N7 from the nitrogen base. Overall, we found 860 interface hydrogen bonds in our training set.

**Table 1:** Hydrogen bonds atomic interaction frequency matrix

		**T**			**C**			**A**			**G**			
					
		**O2**	**N3**	**O4**	**O2**	**N3**	**N4**	**N7**	**N6**	**N3**	**N7**	**O6**	**N2**	**N3**
**R**	**NE**	6	0	3	4	0	0	0	0	0	13	11	0	1
**R**	**NH1**	21	0	2	9	4	0	2	0	8	48	39	0	4
**R**	**NH2**	15	0	8	14	3	0	0	0	7	62	80	0	3
**K**	**NZ**	15	0	9	9	1	0	6	0	7	33	38	0	2
**S**	**OG**	4	0	2	2	0	5	4	1	2	7	9	3	1
**T**	**OG1**	2	1	4	2	0	9	2	1	1	3	3	2	0
**N**	**OD1**	0	1	0	0	0	8	0	23	0	0	0	6	0
**N**	**ND2**	6	0	11	3	0	0	20	0	2	4	11	0	7
**Q**	**OE1**	0	0	0	0	0	4	0	17	0	0	0	4	0
**Q**	**NE2**	2	0	7	3	1	0	16	0	3	1	6	0	5
**H**	**ND1**	1	0	0	0	0	0	0	0	0	2	5	0	0
**H**	**NE2**	3	0	2	1	0	1	0	0	1	5	12	1	1
**Y**	**OH**	1	0	4	3	0	1	1	3	1	2	2	3	1
**E**	**OE1**	0	1	0	0	0	16	0	1	0	0	0	0	0
**E**	**OE2**	0	2	0	0	0	11	0	2	0	0	0	1	0
**D**	**OD1**	0	0	0	0	0	10	0	1	0	0	0	2	0
**D**	**OD2**	0	3	0	0	0	13	0	1	0	0	0	5	0
**C**	**SG**	0	0	1	0	0	0	0	2	0	0	0	0	0
**M**	**SD**	0	0	0	0	0	0	0	0	0	0	0	0	0
**W**	**NE1**	1	0	1	0	0	0	0	0	0	0	0	0	1

Hydrogen bonds reported by HBPLUS [46] between pairs of atoms in the side-chains of amino acids and bases in the set of 210 protein-DNA complexes extracted from PDB were computed. Rows correspond to the hydrogen bond donor/acceptor atoms from the amino acids and columns to the hydrogen bond donor/acceptor atoms from bases (T, C, A, G) with their corresponding PDB atom codes. Each cell indicates the number of contacts found for a given pair of atoms.

In contrast with hydrogen bonds, the landscape of hydrophobic interactions is quite different. As previously reported [9,12], the main discriminatory group regarding specific recognition through hydrophobic interactions is the methyl group of thymine. Accordingly, the C7 group accounted for 20% of all 1010 hydrophobic interactions found in our training dataset, being the main source of contacts for all amino acids when compared with the other nitrogen bases. With the exception of C7, we found no obvious interaction preferences between amino acid and bases. This means that the signal to noise ratio of the calculated preferences is lower than the one observed for hydrogen bonds, and are therefore less informative. Thus, in our subsequent analyses, we decided to take into account only the contribution of the methyl group of thymine during the evaluation of the interface between a TF and a given DNA sequence.

We also generated frequency matrices for water-mediated hydrogen bonds, finding a total of 482 atomic interactions. The overall observed interaction propensities are quite similar to those in the hydrogen bond matrix, which is probably related to the gap filling role of water that supports the existence of long-distance hydrogen bonds [21]. In addition, the buffering role of water allows the formation of electrostatically unfavorable hydrogen bonds, adding new interaction propensities absent in the hydrogen bond matrix. For instance, we found eight water-mediated hydrogen bonds between arginine side-chain nitrogen groups and cytosine N4.

### A survey of the quality of the atomic interaction matrices

The redundancy of the data in the PDB and the bias towards specific protein folds considerably tangle the efforts aiming at exploiting the wealth of this database, limiting the scope of the results. In order to overcome these problems, we decided to avoid redundancy as much as possible in our training set, taking care of withholding a sufficiently informative set of PDB entries. We also planned a thorough bootstrap assay to estimate the quality of the atomic preferences extracted from the training dataset

In this bootstrap tryout we resampled with replacement a thousand subsets of entries, randomly excepting a considerable fraction of the entries included in the initial training set as described in Methods. By leaving out almost a third of the total entries, we measured the degree of overtraining due to residual redundancy in the initial set, as well as the statistical relevance of the interaction preferences reckoned in the matrix building process. A straightforward way of addressing this issue is by finding out whether the matrices constructed using the truncated training sets were good enough to suitably evaluate the direct readout for entries used to build the corresponding bootstrap matrices, though not failing to score the crystallographic complexes that were excluded in the resampling step.

**Figure 1:**
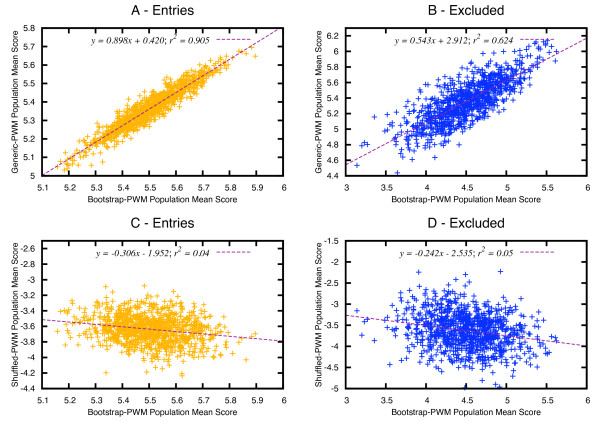
**Scatter plot and regression analysis of the scoring capability of bootstrap matrices against generic and shuffled matrices**. In the top diagrams, the mean score of bootstrap matrices (abscissa) is plotted against the mean score of generic matrices (ordinate) for PDB entries that conform the bootstrapped (A) and excluded (B) datasets. In the bottom graphs, the mean score of bootstrap matrices is plotted against the mean score of shuffled matrices, when computed with entries that are part of the bootstrapped (C) and excluded (D) datasets.

As may be seen in Figure 1, the exclusion of 30% of the entries in the training set does not significantly affect the discrimination capability of the matrices. Here we assess the scores obtained when evaluating the bootstrapped (1A, 1C) and excluded (1B, 1D) datasets with the bootstrap matrices – in the abscissa -, against the generic (1A, 1B) or shuffled matrices (1C, 1D). We found in the scatter plots and the regression analysis that the scores obtained for the assay of the bootstrapped and excluded datasets with the bootstrap matrices correlate very well – i.e., *R*^2 ^= 0.90 and *R*^2 ^= 0.62 respectively -, with the values obtained when assaying those same datasets with the generic matrices (Figure 1A, B). As anticipated, the correlation entirely disappears when the analysis is done with the shuffled matrices – two bottom charts of Figure 1.

### Direct and indirect readout mechanisms in protein-DNA complexes

**Figure 2:**
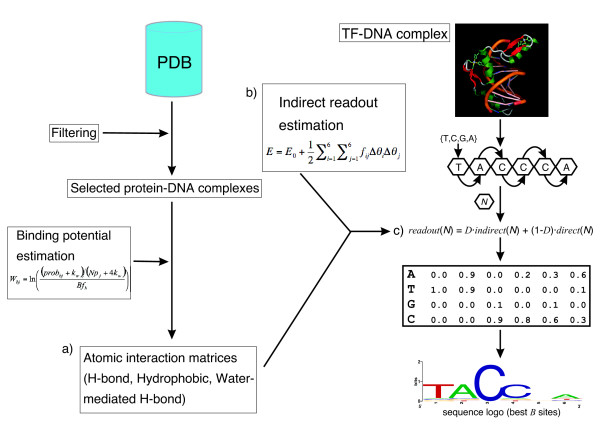
**Flowchart of the DNAPROT algorithm**. Starting from the set of protein-DNA complexes included in the PDB, we filter the entries using the criteria described in the Methods section to eliminate redundancy. a) The culled training set is used to derive atomic matrices that capture the interaction preferences at binding interfaces. Taking as input the Cartesian coordinates of a TF-DNA complex with *N *complementary base pairs, DNAPROT mutates one by one all 4*N *nucleotides in the template. c) During the saturating mutation assay each mutation is scored in terms of direct – i.e., using the atomic PWMs built in step a) – and indirect – i.e., by estimating the deformation cost of DNA upon mutation, as described in step b) – readout and the combined scores are used to fill a position weight matrix. A sequence logo might be calculated from the structure-based PWM by stacking the best *B *oligonucleotides, usually 50.

The information embodied in the atomic interaction matrices presented in the previous section can be used to estimate the contribution of direct readout in protein-DNA recognition. However, it has been shown that a mechanism of indirect readout also plays a relevant role in this process, at least for some TFs, and was therefore considered in this work. In particular, we modeled indirect readout as the cost of threading a nucleotide sequence into a fixed DNA backbone. In order to integrate both direct and indirect recognition mechanisms, we designed a saturating mutation strategy, which is the kernel of the DNAPROT algorithm. Briefly, the algorithm iteratively evaluates the interaction potential of a given TF as the docked nucleotide sequence mutates in a 4*N *space, for a DNA duplex of length *N *(see Figure 2 for a flowchart of the algorithm). This renders a structure-based position weight matrix that can be used to scan genomic sequences. A full description is provided in the Methods section, but it is important to note that direct and indirect readout scores are linearly combined by means of a deformation weight, *D*. As *D *gets larger, the relevance of indirect readout increases.

**Figure 3:**
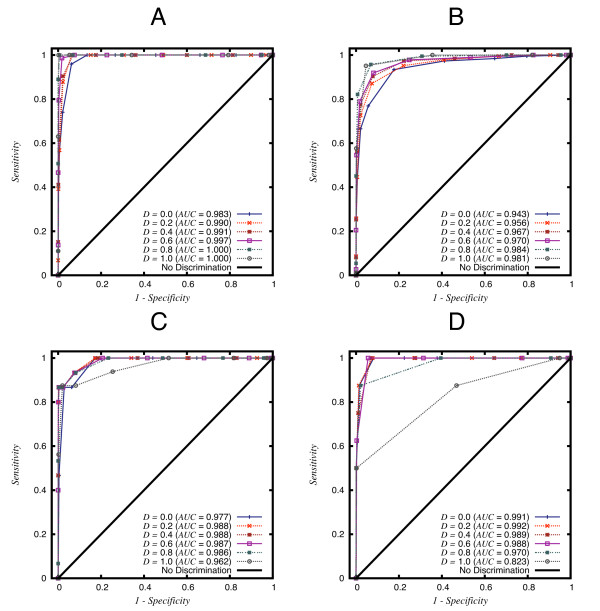
**ROC plots of cognate site recovery in a set of random sequences for NarL, CRP, PurR and DnaA**. Sensitivity (ordinate) and corrected specificity (abscissa) for the cognate binding sites recovery are plotted against in an assay in which the cognate binding sites are rescued from a dataset of non-sense random sequences, as explained in the Methods section. In this assay the *W *parameter is fixed to 1 while *D*, the linear weight of deformation costs assigned to indirect readout, is variable over its domain in the ROC plot analysis for NarL (A), CRP (B), PurR (C) and DnaA (D). In these charts the Area Under the Curve (AUC) is reported for each curve, corresponding to different assays with variable values of *D*. The PDB identifiers are NarL [1je8], CRP [1cgp], PurR [2pua] and DnaA [1j1v].

A study of the performance of our method is shown in Figure 3, where it can be observed that the indirect readout contribution to the ability of correctly scoring cognate sites of CRP and NarL is critical, since the predictive competence increases with large *D *values, as revealed by the increasing Areas Under the Curve (AUCs) in the ROC plots. These outcomes agreed with previous reports in the literature claiming the central role of deformation energy in the site recognition mechanism of CRP [33] and NarL [34], which bend DNA 90° and 42° respectively. PurR and DnaA display an opposite trend, since their largest AUCs are obtained with *D *values less than 0.4. While PurR is known to bend DNA with an angle of 45° [35], the reported bending angle of DnaA is just 28° [36]. This means that although in the first two cases the contribution of deformation can be considered essential, in the cases of PurR and DnaA optimal predictive values are obtained with intermediate values of *D*, also including an important contribution of direct readout.

The subtle changes observed for the AUC in the ROC plots of Figure 3, in fact correspond to significant variations in the classification performance of our method. This can be seen in the results reported in Table 2 for the scoring of PurR known binding sites. The comparison of the PWMs obtained with *D *values of 0.4 and 1.0, those for which we obtained the best and worst performance in Figure 3C, reveals that setting *D *to its optimum value causes the *p*-values of cognate sites to be reduced by one or two orders of magnitude. This improvement corresponds to a reduction of 10^3^-10^4 ^false positives extracted along with true sites in a search set of 10^6^bp. It is worth noting that, using the optimum *D *value in the construction of the model for this TF and setting the sensitivity to 80%, the number of false positive sites recovered with the true sites is as low as 190 in a genome-sized search set.

**Table 2:** Scoring of PurR cognate binding sites using DNAPROT

	***D *= 1.0**		***D *= 0.4**	
**Binding Site**	**Score**	***p***-**value**	**Score**	***p***-**value**
ACGAAACCGTTTGCGT	3.93	1.49E-04	5.68	6.42E-06
ACGAAAACGTTTGCGC	3.84	1.98E-04	5.65	7.02E-06
GCGGAAACGTTTTCCT	3.37	8.15E-04	5.48	1.16E-05
AGGAAAACGGTTGCGT	3.26	1.11E-03	5.41	1.42E-05
CGGAAAACGTTTGCGT	3.22	1.24E-03	5.40	1.46E-05
AAGAAAACGTTTGCGT	3.13	1.59E-03	5.27	2.12E-05
TTGAAATCGTTTGCAT	2.51	7.73E-03	5.06	3.81E-05
ACGCACACGTTTGCGT	3.75	2.62E-04	4.80	7.69E-05
AGGCAAACGTTTACCT	3.27	1.08E-03	4.58	1.37E-04
ACGCAAACGATTACCT	3.00	2.26E-03	4.41	2.10E-04
GCGTAACCGATTGCAT	2.91	2.87E-03	4.37	2.32E-04
TCGCAAACGTTTGCTT	2.80	3.80E-03	4.35	2.44E-04
GAGCAAACGTTTCCAC	2.26	1.37E-02	4.14	4.08E-04
TCGTTCTCTTTTGCCT	0.80	1.92E-01	1.77	4.22E-02
CGGCCAGTTTTTGCAG	-0.43	7.45E-01	0.35	2.58E-01

The known binding sites of PurR reported in RegulonDB [49] were scored using DNAPROT, maintaining *W *fixed to 1 and setting *D *to 1.0 and 0.4. These values of *D *correspond to the parameterization that renders the worst and the best PWMs for this transcription factor respectively. The first column corresponds to the binding sites, the second and fourth columns report the scoring obtained with DNAPROT and the third and fifth columns show the *p*-values of the scores obtained in a site recovery assay in a background set of 10^6 ^random sequences.

### Scoring crystallographic protein-DNA complexes with the DNAPROT algorithm

In this section we present the results of scoring a diverse collection of protein-DNA complexes solved by X-ray crystallography. The test set includes eight transcription factors bound to cognate DNA sequences, of which four are prokaryotic and the other four eukaryotic, belonging to eight different superfamilies according to SCOP [37]. In this experiment we took the coordinates of each of these interfaces and compute structural position weight matrices (PWMs) that approximate their binding specificity. Two types of PWMs are calculated here: *i*) readout matrices, derived using the DNAPROT algorithm outlined in Figure 2, and *ii*) cumulative contact matrices, obtained by simply counting the contacts between protein side-chains and nitrogen bases, as previously described by Morozov and Siggia [32].

**Figure 4:**
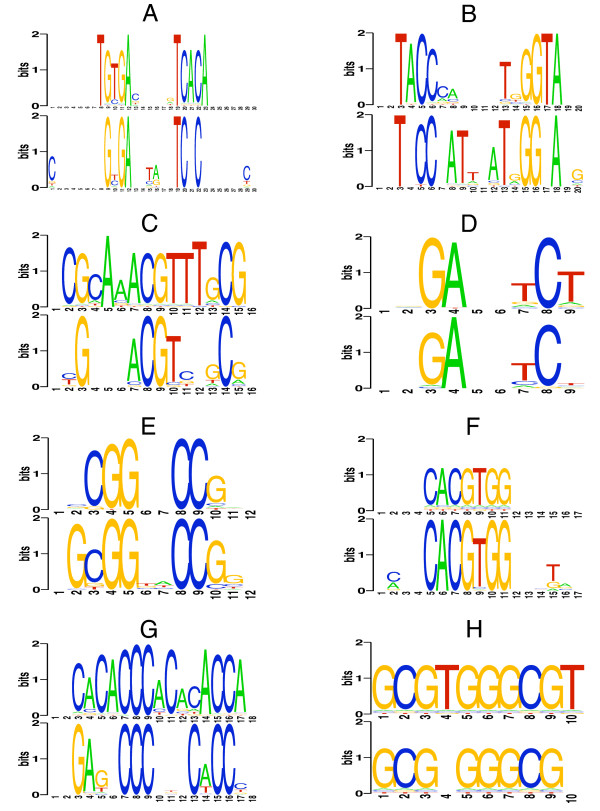
**Stacked sequence logos for PWMs derived from cumulative contact (top) and readout position weight matrices (bottom)**. Four prokaryotic (A: CRP, B: NarL, C: PurR, D: MetJ) and 4 eukaryotic (E: LEU3, F: PHO4, G: RAP1, H: ZIF268) transcription factors were included. See Table 3 for more details.

These PWMs can then be compared with cognate matrices built from experimentally determined binding sites, by means of local alignments [38]. Further details are provided in the Methods section, but it is relevant to note that readout PWMs are computed using knowledge-based atomic potentials, whereas cumulative contact PWMs are calculated with the assumption that the consensus DNA sequence is part of the PDB complex. Figure 4 includes the sequence logos derived from the computed PWMs; the expectation values of PWM comparisons are shown in Table 3, together with details of the test set.

**Table 3:** Comparison of cumulative contact and readout position weight matrices for 4 prokaryotic (top) and 4 eukaryotic (bottom) transcription factors

**SCOP v1.73** **superfamily**	**TF [PDB *id*]**	**Resolution (Å)**	**R_obs_**	***E*-value_contacts_**	***E*-value_readout_**
Winged helix	CRP [1cgp]	3	0.24	7.93E-03	3.76E-05
C-terminal domain of the bipartite response regulators	NarL [1je8]	2.12	0.23	3.58E-05	7.01E-07
					
lambda repressor-like DNA-binding domains	PurR [2pua]	2.9	0.16	4.33E-15	5.51E-01 (7.58E-04)
					
ribbon-helix-helix	MetJ [1cma]	2.8	0.22	1.22E-01	3.30E-01
Zn2/Cys6 DNA-binding domain	LEU3 [2er8]	2.85	0.23	4.97E-06	5.52E-05
HLH, helix-loop-helix DNA-binding domain	PHO4 [1a0a]	2.8	0.23	3.57E-07	3.97E-07
					
Homeodomain-like	RAP1 [1ign]	2.25	0.21	5.52E-03	1.89E-02 (6.40E-04)
C2H2 and C2HC zinc fingers	ZIF268 [1aay]	1.6	0.19	7.93E-14	1.99E-14

In the first column we include the name of the structural superfamily of each TF according to SCOP [37]. In the second column the name of each TF is followed by the PDB identifier of the structure used enclosed in brackets. The following two columns contain the resolution and the *R*-value of the crystals respectively. The last two columns report the *E*-values resulting from STAMP [38] local alignments between the structure-based PWMs obtained with the method of Morozov and Siggia [32] (*E*-value_contacts_) or our approach (*E*-value_readout_) and the corresponding cognate PWMs, extracted from the literature [43,49,53]. Parenthesized *E*-values were calculated after sampling interface rotamers with DNAPROT.

The PWMs generated by DNAPROT starting from crystallographic structures are comparable or better than those produced with the methodology of cumulative contacts by Morozov and Siggia [32]. This is clear in the results of Table 3, where, with the exception of PurR and LEU3, the statistical significance of our readout matrices is higher or of the same order as that of the contact matrices. An inspection of Figure 4 also shows that for the three prokaryotic regulatory proteins, CRP, NarL and MetJ, the sequence logos obtained by both methods are quite similar. However, except for the case of MetJ, for which the significance of the PWMs generated by both methods is of the same order of magnitude, for CRP and NarL the matrices generated by our algorithm are two orders of magnitude better. On the contrary, although the logo of PurR is correct, the *E*-value it gets when compared with the expected logo is several orders of magnitude worse, as a consequence of failing to identify 5 consensus nucleotides. The results for our selection of eukaryotic TFs show close similarities for the statistical significance of the PWMs generated by both methods. However, for RAP1 sampling of interface rotamers caused an improvement of the readout matrix that outperforms the contact matrix by one order of magnitude, (see Table 3 for parenthesized *E*-values). Something similar happened for the matrix generated for PurR, though in this case the improvement of three orders of magnitude of the significance is still worse than the value of the contacts PWM.

### Footprinting comparative models of protein-DNA complexes with DNAPROT

In this section we further test the ability of DNAPROT readout matrices to capture the binding specificity of TF-structural models. Here we generated homology models for two transcription factors: FNR, a global regulatory protein in *E. coli*, and Giant, a regulatory protein involved in the early development of *D. melanogaster*. As in the previous section, we also computed cumulative contact PWMs for comparison purposes, and we evaluated both structure-based matrices by aligning them to cognate matrices built from operator sites reported in the literature. We now present the results of both models in more detail.

**Figure 5:**
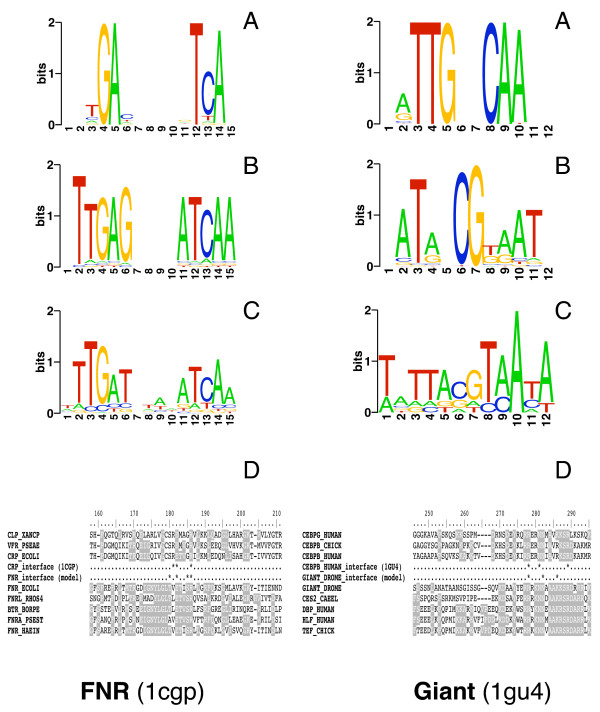
**Stacked sequence logos for comparative models of FNR and Giant**. A: cumulative contact logo. B: readout logo. C: logo derived from cognate sites reported in the literature. D: multiple sequence alignment of DNA-binding interface of homologous sequences for the modeled sequences and the templates used in modeling. Residues marked with asterisks participate in specific DNA recognition. See Table 4 for more details.

The protein sequence of FNR can be confidently aligned to the sequence of *E. coli *CRP (PDB *id*: 1cgp; alignment coverage = 71%) with TFmodeller [39], despite an overall sequence identity of just 22%, providing a reliable frame for comparative modeling. However, several residue substitutions occur at the binding interface, suggesting that the readout might be changing from the template to the model. Indeed we found that four out of ten interface residues are mutated in the dimeric model, as annotated in Table 4. For this reason we sampled different rotamers for the side-chains of the interface residues and selected those that yielded best readout scores using the DNAPROT algorithm. As shown in Figure 5, this limited sampling was enough to improve the readout PWM, increasing the number of correct consensus nucleotides in the sequence logo from six to nine. This improvement of the model is also related to an increase of the statistical significance of the readout matrix of one order of magnitude with respect to the cumulative contact matrix, as shown in Table 4. A further improvement is also possible by deriving a contact matrix from the refined homology model, which causes an additional increase of the *E*-value to 1.46E-04. The multiple alignment, Figure 5D, also suggests that the interface differences inferred from the comparative model correlate with the sequence conservation of the DNA-binding domain among FNR and CRP homologous sequences.

**Table 4:** Comparison of cumulative contact and readout position weight matrices derived from two comparative models for the FNR and Giant transcription factors

**SCOP v1.73** **superfamily**	**TF [PDB *id*]**	**Interface** **identity**	**Resol**. **(Å)**	**R**_obs_	***E***-**value**_contacts_	***E***-**value**_readout_
Winged helix	FNR [1cgp]	6/10	3	0.24	1.40E-01	6.03E-01 (6.35E-02) [1.46E-04]
Leucine zipper domain	Giant [1gu4]	6/12	1.8	0.23	7.10E-03	3.65E-02 (4.52E-04)

In the first column we include the name of the structural superfamily of each TF according to SCOP [37]. The name of each model is followed by the PDB identifier of the template used to guide model building, enclosed in brackets. The following three columns contain the ratio of contact interface identity, the resolution and the *R*-value of the crystals respectively. The last two columns report the *E*-values resulting from STAMP [38] local alignments between the structure-based PWMs obtained with the method of Morozov and Siggia (*E*-value_contacts_) or our approach (*E*-value_readout_) and the corresponding cognate PWMs, extracted from the literature [49,54]. Parenthesized *E*-values were calculated after sampling interface rotamers while bracketed *E*-values correspond to cumulative contact PWMs computed over the refined DNAPROT complex.

The second study of a homology model was performed with Giant, an eukaryotic leucine zipper transcription factor that has been studied previously [24]. This protein can be modeled based on human CEBPB (PDB *id*: 1gu4), with a sequence alignment that covers 92% of the DNA-binding domain. Although the overall sequence identity is only 13%, six out of 12 interface residues, as labeled by TFmodeller, are conserved. The first evaluated Giant model yielded a PWM that captures only four correct consensus positions, as seen in Figure 5. As in the case of FNR, we refined the binding interface of Giant by sampling rotamers and selecting those with best readout scores. The refined model yields a structure-based PWM that includes eight consensus nucleotides, improving the preliminary model results in two orders of magnitude in terms of *E*-value (see Table 4). This sampled model also suggests a different set of interface residues responsible for DNA sequence discrimination. The figure shows an alignment of Giant and CEBPB homologous sequences that seem to cluster in two different modes of binding to DNA.

### Relative contribution of atomic interactions at DNA recognition interfaces

The DNAPROT algorithm models the direct readout mechanism by labeling hydrogen bonds, hydrophobic interactions and water-mediated hydrogen bonds at the interface, which are evaluated by means of *log*-likelihood preference matrices. Therefore, the data generated for the eight TFs presented in Table 3 can be further analyzed with the aim of calculating the relative contribution of each of these atomic interactions to DNA recognition. As shown in Table 5, we found four cases in which recognition is mediated exclusively by hydrogen bonds (CRP, LEU3, PHO4 and RAP1), while the remaining TFs seem to employ two or more types of atomic interactions to drive DNA binding. Of these, perhaps the most interesting cases are NarL, which has a large hydrophobic contribution, and ZIF268, with 30% of the interface score contributed by water-mediated hydrogen bonds. Repeating these calculations on the set of non-redundant complexes, we found that 78% of DNAPROT interface scores are contributed by hydrogen bonds, while water-mediated hydrogen bonds account for 16% and hydrophobic interactions are responsible for 6% of the total. Table 5B shows a more detailed analysis of NarL and ZIF268, in which structure-based PWMs derived from only one type of atomic interactions are compared with the same cognate PWMs in Table 3. These last results show that the combination of explicit representations of the relative contribution of the different types of interaction improves the predictive competence of DNAPROT.

**Table 5:** Contribution of hydrogen bonds, hydrophobic contacts and water-mediated hydrogen bonds to specific DNA recognition in terms of interface binding score (A) and STAMP E-value of structure-based PWMs (B)

**A) Fraction of interface binding score contributed by:**				
	**H-bonds**	**Hydrophobic contacts**	**Water-mediated** **H-bonds**	**# atomic interactions**
CRP	1.00	0.00	0.00	8
PurR	0.76	0.24	0.00	6
MetJ	0.89	0.00	0.11	6
NarL	0.61	**0.34**	0.05	11
LEU3	1.00	0.00	0.00	4
PHO4	1.00	0.00	0.00	7
RAP1	1.00	0.00	0.00	12
ZIF268	0.70	0.00	**0.30**	21

**B) *E*-value of PWM derived from:**				

	**H-bonds**	**Hydrophobic contacts**	**Water-mediated** **H-bonds**	**All atomic interactions**
NarL	1.049E-02	**4.837E-04**	-	7.010E-07
ZIF268	1.359E-11	-	**6.957E-05**	1.998E-14

A) Transcription factors in Table 3 where further analyzed by assessing the fraction of the interface binding score, obtained after summing all *log*-likelihood atomic scores, contributed by each interaction type. The number of atomic interactions found by the DNAPROT protocol in the native PDB structure is indicated in the rightmost column. B) A further analysis of two notable examples, NarL with an important hydrophobic contribution and ZIF268 with a remarkable contribution of water-mediated hydrogen bonds, in which structurally derived PWMs that consider exclusively H-bonds, hydrophobic contacts or water-mediated H-bonds are compared with default DNAPROT PWMs, which integrate all three atomic interactions.

## Discussion

The field of computational prediction of regulatory sites in genomic sequences has been dominated by methodologies relying on prior knowledge of experimentally characterized binding sites [6,40]. Only a limited group of studies has intended to generate structural representations of the sequence recognition interface, using approaches that somehow approximate the physical processes involved in DNA recognition [9,24,25]. Some of these studies have dissected binding interfaces at the residue level [9,13,27,41], while others use atomic-based descriptions [24,25,42]. Though some efforts employ force fields for the calculations, most of these methodologies rely on pre-compiled interaction preferences. Moreover, there are also conceptually simpler approaches that rely on counting contacts at the interface [16,17,32] that generally work under the assumption that the nucleotide sequence captured in a crystallographic protein-DNA complex is the consensus. These contact-based approaches, particularly the cumulative-contact method of Morozov and Siggia, promise to be successful in binding site searches at the genomic scale if the structure of a bound TF is at hand.

### Extraction of protein-DNA atomic interaction preferences from the PDB

The DNAPROT algorithm introduced here incorporates an atom-based representation of protein-DNA interfaces and explicitly integrates both direct and indirect readout. Though the thermodynamic contribution of interface desolvation and conformational entropy of interacting groups have been considered in some approaches using complex formulations [23,24], our algorithm, outlined in Figure 2, is concerned with binding specificity and does not account for those contributions, like other related reports [18]. However, our methodology attempts to approximate these phenomena by including explicit representations of binding interface water molecules and side-chain rotamer sampling of interacting amino acids, which have important implications in our results.

The hydrogen bond and hydrophobic interaction matrices used by DNAPROT were derived from a non-redundant set of 210 complexes, naturally surpassing the training sets used in preceding studies, as more structural data is now available at the PDB. An important assumption behind the construction of this data set is that rules governing specific recognition of DNA by proteins are generally due to the conformational restrictions imposed by the double helix. Therefore, we chose to collect a comprehensive training set instead of using a superfamily-specific or a TF-exclusive set, in which the scantiness of information would have weakened the derived statistical models. In addition, we built an atomic contact matrix that explicitly accounts for water-mediated hydrogen bonds in protein-DNA interfaces, which constitutes a novel contribution of this work. The data obtained in the construction of our contact propensity matrices, such as the hydrogen bond matrix in Table 1, constitute an update of the pioneer work by Mandel-Gutfreund *et al*. [10] and Luscombe *et al*. [12] as we found similar trends in the atomic interaction propensities in our enlarged dataset. In addition, this work presents quantitative arguments (see Table 5 for details), which demonstrate the importance of including hydrophobic and water-mediated hydrogen bonds in the list of interactions that contribute to specific recognition of nucleotide sequences.

### Assessment of the quality of the atomic interaction matrices

Keeping in mind the problem of data bias in the PDB, we planned an in-depth bootstrap tryout to assess the reliability of the information contained in our contact matrices. The exclusion of almost a third of the entries in the original training set yielded atomic interaction matrices with only a slightly reduced ability to correctly evaluate infrequent atomic contacts. These results confirm that the preference matrices derived in this work are biologically significant and that the atomic associations found are not significantly affected by overtraining nor by redundancy, since bootstrap matrices scored the excluded training sets almost as well as the bootstrapped ones. On the contrary, the results for the negative control obtained while repeating this analysis with shuffled matrices (two bottom charts in Figure 1), show completely scattered distributions with no correlation. These outcomes support the consistency of the interaction preferences extracted from the training set used to build our matrices. They also corroborate that the inclusion of new protein-DNA complexes, regularly deposited in the PDB, will not cause considerable variations of the interaction preferences, except for very unusual atomic contacts.

### Evaluation of direct/indirect readout contributions to protein-DNA recognition

The rationale for using atomic potentials is that many interface contacts are either complex or bidentate [12], and therefore cannot be properly accounted for by residue-based approaches. In those cases the contact pairing ratio is not 1:1, as the same amino acid might be in contact with two base steps simultaneously or binding a given nitrogen base through multiple groups. These drawbacks could be resolved by using explicit atomistic representations of the mode of binding, which have been the goal of a group of papers in this field [18,25,32]. However, using atomic potentials requires high-quality atomic structures of protein complexes. In addition, atomic-detail approaches such as DNAPROT might be more affected by cases in which side-chains rearrange upon mutation of the bound nucleotides [43], which is the reason that it might be necessary to sample interface rotamers while doing comparative modeling, as explained in the Results section. Further sampling, including backbone movements, might be required in certain cases, as already envisaged by Havranek *et al*. [23]. In fact, previous works have already recognized that homologous protein-DNA interfaces change their docking geometry as their sequences diverge [30,44].

Besides the contribution of the direct readout to sequence recognition, we also considered in our model a mechanism of indirect readout, as sequence-specific DNA deformation has been identified as key to DNA recognition for many transcription factors [33,34]. The algorithm presented in this work follows previous efforts that model indirect readout as the cost of deforming a DNA duplex [13,14,24]. Both readout mechanisms, shown to be critical for specific recognition in Figure 3, are linearly integrated into a single binding potential. The weights of both direct and indirect readout can be tuned for different transcription factors according to their docking mode, as the examples depicted in Figure 3 imply. This observation suggests that the performance of the DNAPROT protocol can be improved using previous biological knowledge and this is certainly a desirable property. This data also insinuates that each TF might have its own balance between direct and indirect readout, although we cannot exclude the possibility that this value is a property of its structural superfamily. Moreover, data in Table 2 gives further insight into the impact that correct weighting could have on genomic scale TF-binding-site prediction assays, as the number of false positives might be considerably reduced in order to obtain a more reliable set of predictions.

### Assaying the predictive potential of DNAPROT in crystallographic structures and homology models

The predictive power of DNAPROT, evaluated in Tables 3 and 4 and Figures 4 and 5, suggests that our readout PWMs are, with one exception, comparable to or better than those generated by a well established reference methodology, the cumulative contact PWM proposed by Morozov and Siggia [32] and related to previous reports [16,17]. It is worth remembering the reader that, unlike the reference method, DNAPROT does not assume that the nucleotide sequence in the input PDB complex is the consensus; rather, it performs an *in silico *mutagenesis assay and evaluates 4*N *sequences using a readout function. As already mentioned, the conceptually simpler cumulative contact approach does not consider the contribution of indirect readout. This might explain the better results obtained with our method for CRP and NarL shown in Table 3, two TFs known to have an important contribution of indirect readout [33,34]. In contrast, the example of PurR shows that the DNAPROT formulation of hydrogen bonds and hydrophobic interactions does not fully capture the array of atomic contacts at the interface, as the obtained readout sequence logo misses 4 consensus positions that might correspond to a different type of interaction. Despite this fact, the predicted PWM correctly identifies the remaining consensus positions except one, and the statistical significances obtained in the site recovery assay of the cognate binding sites of this TF depicted in Table 2 prove the predictive competence of the matrix built.

Notwithstanding the promising results obtained for crystallographic structures, the most valuable application of the methodology presented in this paper is found in the exercise of comparative modeling. Two examples were modeled here, a well-characterized prokaryotic regulator (FNR) and an eukaryotic factor previously studied in a related article [24]. Both examples demonstrate that rotamer sampling at the interface is necessary for obtaining optimal results, and we found that the readout formulation presented here can be effective in selecting the best rotamers. As shown in Table 4 and Figure 5, our methodology yielded better results than those obtained with the cumulative contact method for two homology models. The reason for this could be that comparative models usually contain errors in the assigned position of protein side-chains and, most importantly, they do not necessarily contain the consensus DNA sequence. A combination of *in silico *saturating DNA mutagenesis and interface side-chain sampling allows the DNAPROT algorithm to partially overcome these problems. Not only is sampling positive in modeling tasks, but we also found that the crystallographic structures of PurR and RAP1 yielded better readout PWMs after resampling their interface side-chain rotamers.

Although the quality of the structures used by our methodology is of primary relevance, we could not find a clear correlation between R-factor, resolution or experimental techniques for entries in the PDB and the outcomes of our procedure. Nevertheless, we often encountered protein-DNA complexes where only a few hydrogen bonds or hydrophobic interactions can be identified using standard bond geometries, yielding only partial DNA motifs. In such cases we found that the cumulative contact method seems to be less sensitive to the quality of structural data. Further work needs to be done to explore whether side-chain sampling, and even backbone sampling, can help circumvent the limitations that data quality imposes on the performance of readout PWMs.

### Evaluation of the contribution of different interaction types to the recognition process

The atomistic foundation of our methodology also gives us the possibility of exploring the relative contribution of each interaction type to the DNA recognition process. The analysis of a non-redundant set of more than 200 complexes proves that water-mediated hydrogen bonds are the second source of specific interactions at contact interfaces, which raises a warning regarding the usual exclusion of these interactions in structural studies trying to model protein-DNA interaction interfaces. The exclusion of water overlooks a wide group of highly informative contacts, mainly long-distance and unfavorable hydrogen bonds [21] that constitute novel pairings absent from the hydrogen bond preference matrix. The example of ZIF268 analyzed in Table 5B, shows that an important part of the information content of the PWM obtained for this TF corresponds to water-mediated interactions, and the inclusion of this information considerably increases the statistical significance of the readout matrix. Something similar occurs for hydrophobic interactions; despite being relatively infrequent sometimes their contribution to binding might be central. This is the case of the matrix obtained for NarL, in which hydrophobic links account for most of the information content of the structurally-derived PWM, despite being the least frequent interface atomic interaction. In this last example, without a reliable inclusion of those infrequent interactions, our model for this TF would have been worthless.

## Conclusion

In summary, our results suggest that the DNAPROT algorithm, together with the set of atomic interaction matrices obtained in this work, have useful applications. The matrices contain biologically meaningful information that confirm and enrich previous reports at the atomic level of interaction. In addition to the uses presented in this paper, these matrices could be taken to estimate specificity of binding [45] or as a guide for crystallographic studies of protein-DNA complexes. With respect to the algorithm, previous work by Morozov and Siggia [32] demonstrated that cumulative contact PWMs could be used as informative priors in the task of scanning genomic sequences. In this work we found that our algorithm outperforms the aforementioned method for homology models of TFs and displays a comparable performance with crystallographic structures. This fact gives relevance to the statistical models that can be generated with DNAPROT and preliminary tests while scanning genome-sized sequence sets with the model built for PurR confirm this expectation. Overall, our study adds new insights to the challenging problem of estimating protein-DNA binding specificities from structural complexes alone.

## Methods

### Construction of frequency and weight matrices for protein-DNA atomic interactions

A set of 210 protein-DNA complexes was extracted from the Protein Data Bank [31] (release February 29^th ^2008) and was used to compute the atomic preferences of interaction between proteins and DNA that drive specific recognition. All these complexes were solved by X-ray crystallography to a resolution ≤ 3 Å. We started from the weekly updated clusters of sequence similarity disclosed as part of the PDB derived data, rejecting entries with protein chains shorter than 30 amino acids or DNA chains shorter than 10 bases. Entries having less than four Cα within 12 Å from atoms N1/N9 of nitrogen bases were also excluded. Whenever multiple entries of the same protein were found, the one with the best resolution was taken.

To circumvent redundancy in the estimation of atomic contact preferences we considered only protein chains from complexes having a sequence identity less than 50% than any other protein chain in the dataset. This cut-off approximately marks the point at which the geometry of the contact interface of protein-DNA complexes with similar amino acid sequence start to diverge [30]. A second filter was used to remove complexes with more than 70% atomic contacts identical to other complexes from the same SCOP [37] superfamily. This is necessary because members with a percentage of sequence identity in the boundaries of the 50% cutoff can still have very similar DNA interfaces – i.e., with similar structural geometries and involving the same atoms. As the number of atomic interactions in protein-DNA complexes tends to be small, this second filter effectively removes identical or nearly identical interfaces within superfamilies.

Hydrogen bonds, water-mediated hydrogen bonds and hydrophobic contacts were calculated using a modified version of the program HBPLUS [46]. We set the input parameters of the program to the default distance and angle restriction for the estimation of hydrogen bonds (H-A distance < 2.7 Å; D-A distance < 3.35 Å; D-H-A angle > 90° and H-A-AA angle > 90°, being AA the atom attached to the hydrogen acceptor atom). For hydrophobic contacts we considered atoms CB, CG, CG1, CG2, CD1, CD2, CE and CZ in proteins and all carbon atoms of nitrogen bases, including only contact distances in the range of 3.0–3.9 Å in order to exclude sterically impossible pairings. This resulted in three atomic frequency matrices such as the one reported in Table 1 (refer to Additional file 1 for the matrices generated for hydrophobic and water-mediated hydrogen bonds and a complete list of the PDB entries used to construct them).

The atomic contact specific frequency matrices were converted to weight matrices by using a modified version of the expression described by Hertz and Stormo [5]:

$$W_{ij}=\ln\frac{\left(n_{ij}+p_{ab}\right)/\left(N_{a}+1\right)}{p_{ab}}$$

where *W*_*ij *_is the *log*-likelihood interaction probability of atom *i *from the amino acid and atom *j *from the nitrogen base, *n*_*ij *_is the number of specific contacts observed for atom *i *of the amino acid and *j *of nitrogen base. In addition, *p*_*ab *_is the number of expected contacts for amino acid and base given their correspondent abundance in the training dataset normalized by the number of donor/acceptor atoms both in the amino acid and nitrogen base, and *N*_*a *_is the total number of contacts observed for amino acid *a *with all the four nitrogen bases. Matrices of this kind assume complete independence for the statistical contribution of each position to the final score of the sequence, which correspond to the simplest and more popular model of PWMs [5,6]. Using this model the partial contributions of all interacting atoms may be summed up to approximate the binding energy of the site. By taking into consideration hydrophobic and hydrogen bond contacts, we can infer the direct readout interaction potential of a given DNA sequence threaded into a crystallographic complex. Note that there are other possible ways to account for atomic contacts [42].

### Bootstrap analysis of the training set used in the construction of the atomic interaction matrices

Starting from the 210 PDB entries in the initial set, we randomly sampled 1000 partial training sets excluding 30% of the entries. Accordingly, a thousand "bootstrap" matrices for hydrogen bond, water-mediated hydrogen bonds and hydrophobic contacts were generated, which were in contrast to three "generic" matrices – i.e., those built with all entries. These bootstrap matrices were used to evaluate the binding interface of all the PDB entries in the partial training set as well as the entries of the excluded dataset – i.e., those that were randomly excluded in the sampling process. Interface scores were normalized by the number of protein-DNA contacts in the entry. In order to follow the progress of the experiment a population mean value was computed for each bootstrapped and excluded dataset.

As a negative control, we used randomly shuffled matrices constructed from the generic matrices, in which the value corresponding to the interaction probability of a particular pair of atoms is randomly interchanged with any other from the matrix. The final shuffled matrices retained the general characteristics of the generic matrices – e.g., the information content and the maximum possible score for the consensus sequence – but have lost all the information about the amino acid-nitrogen base atomic interaction preferences extracted when using the original dataset.

### The DNAPROT algorithm for sequence threading and estimation of interaction potential

DNAPROT is a computer program designed to thread DNA sequences into the structure of a given protein-DNA complex with the purpose of estimating their interaction potential. DNAPROT is written in C++ and PERL and makes use of 3DNA [47] and a modified version of HBPLUS [46]. Threading is performed by substituting the nitrogen bases of nucleotides found in the crystallographic complex – which can be tentatively termed as "native" – by those in the input sequence, maintaining the coordinates of the sugar/phosphate backbone. The coordinate system used for base substitution is the same described by Kono and Sarai [9] in which the origin is the N1/N9 atom, preserving the planarity and orientation of nitrogen rings.

In order to estimate the interaction potential between a threaded DNA sequence and the docked protein, we used a straightforward strategy to account for the relative contributions of direct and indirect readout. The direct readout is related to the set of hydrogen bonds (as defined by HBPLUS) or hydrophobic interactions existing among amino acids and bases at the major and minor groves. This contribution can be computed using the atomic preference matrices described above in this Method section, summing up the likelihood of each pair of interacting atoms. The indirect readout can be approximated as the cost of threading a nucleotide sequence into a DNA backbone with fixed geometric step parameters – i.e., step, shift, slide, rise, tilt, roll, twist -, calculated from the original coordinates by 3DNA. For this calculation we used the harmonic function and the set of experimentally-derived spring parameters described by Olson *et al*. [48], as in the work by Morozov *et al*. [24].

In order to derive a structure-based position weight matrix, DNAPROT performs an *in silico *"saturation mutagenesis" of the native DNA chain replacing the native nucleotide found in a given position by the other three possible bases, preserving the sequence of the other positions in the binding site. Each mutated sequence generated by this procedure is evaluated combining both direct and indirect readout scores, which are converted to a probability using the following expression, inspired by previous work by Morozov *et al*. [24]:

$$prob_{bj}=e^{-(\left(1-D\right)\times direct_{bj}+D\times indirect_{bj})}$$

where *D *is a linear weight used to ponder the relative direct and indirect readout contributions, taking values from 0 to 1 (0.5 by default), and *direct*_*bj *_and *indirect*_*bj *_are the direct and indirect readout scores calculated for the substitution of base *b *in position *j *of the DNA sequence found in the crystallographic complex. The iteration of this column-wise procedure for all the positions of the original sequence generates a position weight matrix embodying information from both the direct and indirect contributions. The elements of this PWM are calculated using the following equation:

$$W_{bj}=\ln\left(\frac{\left(prob_{bj}+k_{w}\right)/\left(Np_{j}+4k_{w}\right)}{Bf_{b}}\right)$$

where *W*_*bj *_is the contribution of base *b *in position *j *to the final interaction potential, *prob*_*bj *_is calculated as described above, *Np*_*j *_is the summation of the probabilities for the mutation of the four bases in position *j *of the binding site, and *k*_*w *_is a pseudo-count weight computed as:

$$k_{w}=W\frac{Np_{j}}{4}$$

where *W *is an empirical parameter taking variable values between 0 and 1 (0.01 by default, although our tests suggest that has little effect), which is useful to give the model the possibility of exploring sequence alternatives more or less different from the starting sequence used as model depending on the value of *W*, making it more or less restrictive. *Bf*_*b *_is the background frequency of nitrogen base *b *in the genomic context.

### Benchmark of the predictive methodology with random sequences

In order to evaluate the predictive power of DNAPROT, Receiver Operating Characteristic (ROC) curves were constructed for NarL, CRP, PurR and DnaA, four transcription factors from *E. coli *that have been co-crystallized with a DNA operator site. The number of known sites extracted from RegulonDB [49] that constitute the sets of cognate sites is enclosed in parenthesis after the TF name followed by the PDB entry *id *enclosed in brackets: NarL (73) [1je8], CRP (182) [1cgp], PurR (15) [2pua], and DnaA (8) [1j1v]. A set of approximately 10^6 ^random sequences with the same background %GC content of the *E. coli *genome was generated with the RSA-Tools suite [50].

To construct the ROC curves the sets of cognate and random sequences were threaded into the DNA chain found in the crystallographic complex – i.e., the native DNA sequence – to estimate the interaction potential. The values of sensitivity and specificity were then calculated using the following well-known formulae:

*Sensitivity *= *TP*/(*TP *+ *FN*)

*Specificity *= *TN*/(*TN *+ *FP*)

where *TP *is the number of cognate sites with binding scores equal to or higher than a given cutoff and *FN *is the number of cognate sites which do not pass the cutoff. *TN *is defined as the number of random sequence fragments scoring bellow the score cutoff and *FP *as the number of random sequences fragments passing the cutoff, considered as putative binding sites. As it is a common practice in ROC curve construction, the cutoff values referenced above are recursively assigned during an exhaustive scan of the complete interaction potentials range, from the highest to the lowest value of the union of random and cognate datasets. The area under the curve (AUC) used as a measure of the quality of the classifier was estimated using the trapezoid algorithm.

### Computing cumulative contact PWMs

Cumulative contact position weight matrices were calculated by iterating across all base pairs in the template DNA, and adding all protein side-chain atomic contacts within 4.5 Å of the nitrogen ring. The total number of contacts for each base pair can be translated to a column in the resulting PWM using the formula proposed by Morozov and Siggia [32].

### Comparative modeling of transcription factors

Models for FNR and Giant were constructed using the TFmodeller software . Two input files were provided for each model: *i*) a PDB file with coordinates of the chosen template in dimeric form and *ii*) a sequence alignment of two concatenated copies of the modeled factor and the template in FASTA format. The binding interface of these models was further refined by sampling rotamers using the computer program SCWRL version 2.7 [51] choosing the multiple conformations options. The native DNA sequence of the template was put in place (-f option) to add steric boundaries.

### Comparison of PWMs

The computer program STAMP [38] was used to calculate pairwise Smith-Waterman alignments of position weight matrices, using the default Pearson correlation metric and the precompiled random score distributions.

### Converting PWMs to sequence logos

Sequence logos were generated using WEBLOGO [52] and taking as input the best *B *nucleotide sequences scored by a reference PWM. *B *is set by default to 50, but can in some cases be a smaller number *b*, if the *(b*+1)-*th *site has a score that is worse than the worst single nucleotide consensus mutation.

## Abbreviations

TF: Transcription Factor; PDB: Protein Data Bank; PWM: Position Weight Matrix; ROC: Receiver Operating Characteristic; SCOP: Structural Classification of Proteins; AUC: Area Under the Curve.

## Authors' contributions

VEA, AGP and BCM participated in the conception of the study. VEA and BCM designed and performed the research and drafted the manuscript. ATV and JCV supported the project. AGP, ATV and JCV helped revise the manuscript. All authors have read and approved the manuscript.

## Supplementary Material

Additional File 1 **Excel spreadsheet listing all three atomic interaction matrices described in the paper and the list of PDB complexes used to derive them.** Click here for file

## References

[B1] Takeda Y, Sarai A, Rivera VM (1989). Analysis of the sequence-specific interactions between Cro repressor and operator DNA by systematic base substitution experiments. Proc Natl Acad Sci USA.

[B2] Choo Y, Klug A (1994). Selection of DNA binding sites for zinc fingers using rationally randomized DNA reveals coded interactions. Proc Natl Acad Sci USA.

[B3] Schneider TD, Stormo GD, Gold L, Ehrenfeucht A (1986). Information content of binding sites on nucleotide sequences. Journal of molecular biology.

[B4] Berg OG, von Hippel PH (1988). Selection of DNA binding sites by regulatory proteins. Trends Biochem Sci.

[B5] Hertz GZ, Stormo GD (1999). Identifying DNA and protein patterns with statistically significant alignments of multiple sequences. Bioinformatics.

[B6] Stormo GD (2000). DNA binding sites: representation and discovery. Bioinformatics.

[B7] Mandel-Gutfreund Y, Baron A, Margalit H (2001). A structure-based approach for prediction of protein binding sites in gene upstream regions. Pac Symp Biocomput.

[B8] Suzuki M, Yagi N (1994). DNA recognition code of transcription factors in the helix-turn-helix, probe helix, hormone receptor, and zinc finger families. Proc Natl Acad Sci USA.

[B9] Kono H, Sarai A (1999). Structure-based prediction of DNA target sites by regulatory proteins. Proteins.

[B10] Mandel-Gutfreund Y, Schueler O, Margalit H (1995). Comprehensive analysis of hydrogen bonds in regulatory protein DNA-complexes: in search of common principles. Journal of molecular biology.

[B11] Mandel-Gutfreund Y, Margalit H (1998). Quantitative parameters for amino acid-base interaction: implications for prediction of protein-DNA binding sites. Nucleic Acids Res.

[B12] Luscombe NM, Laskowski RA, Thornton JM (2001). Amino acid-base interactions: a three-dimensional analysis of protein-DNA interactions at an atomic level. Nucleic Acids Res.

[B13] Selvaraj S, Kono H, Sarai A (2002). Specificity of protein-DNA recognition revealed by structure-based potentials: symmetric/asymmetric and cognate/non-cognate binding. Journal of molecular biology.

[B14] Michael Gromiha M, Siebers JG, Selvaraj S, Kono H, Sarai A (2004). Intermolecular and intramolecular readout mechanisms in protein-DNA recognition. Journal of molecular biology.

[B15] Luscombe NM, Thornton JM (2002). Protein-DNA interactions: amino acid conservation and the effects of mutations on binding specificity. Journal of molecular biology.

[B16] Mirny LA, Gelfand MS (2002). Structural analysis of conserved base pairs in protein-DNA complexes. Nucleic Acids Res.

[B17] Raviscioni M, Gu P, Sattar M, Cooney AJ, Lichtarge O (2005). Correlated evolutionary pressure at interacting transcription factors and DNA response elements can guide the rational engineering of DNA binding specificity. Journal of molecular biology.

[B18] Paillard G, Lavery R (2004). Analyzing protein-DNA recognition mechanisms. Structure.

[B19] Mandel-Gutfreund Y, Margalit H, Jernigan RL, Zhurkin VB (1998). A role for CH...O interactions in protein-DNA recognition. Journal of molecular biology.

[B20] Wahl MC, Sundaralingam M (1997). C-H...O hydrogen bonding in biology. Trends Biochem Sci.

[B21] Jayaram B, Jain T (2004). The role of water in protein-DNA recognition. Annu Rev Biophys Biomol Struct.

[B22] Kalodimos CG, Biris N, Bonvin AM, Levandoski MM, Guennuegues M, Boelens R, Kaptein R (2004). Structure and flexibility adaptation in nonspecific and specific protein-DNA complexes. Science.

[B23] Havranek JJ, Duarte CM, Baker D (2004). A simple physical model for the prediction and design of protein-DNA interactions. Journal of molecular biology.

[B24] Morozov AV, Havranek JJ, Baker D, Siggia ED (2005). Protein-DNA binding specificity predictions with structural models. Nucleic Acids Res.

[B25] Robertson TA, Varani G (2007). An all-atom, distance-dependent scoring function for the prediction of protein-DNA interactions from structure. Proteins.

[B26] Benos PV, Lapedes AS, Stormo GD (2002). Probabilistic code for DNA recognition by proteins of the EGR family. Journal of molecular biology.

[B27] Kaplan T, Friedman N, Margalit H (2005). Ab initio prediction of transcription factor targets using structural knowledge. PLoS Comput Biol.

[B28] Sarai A, Siebers J, Selvaraj S, Gromiha MM, Kono H (2005). Integration of bioinformatics and computational biology to understand protein-DNA recognition mechanism. J Bioinform Comput Biol.

[B29] Liu Z, Mao F, Guo JT, Yan B, Wang P, Qu Y, Xu Y (2005). Quantitative evaluation of protein-DNA interactions using an optimized knowledge-based potential. Nucleic Acids Res.

[B30] Contreras-Moreira B, Collado-Vides J (2006). Comparative footprinting of DNA-binding proteins. Bioinformatics.

[B31] Berman H, Henrick K, Nakamura H, Markley JL (2007). The worldwide Protein Data Bank (wwPDB): ensuring a single, uniform archive of PDB data. Nucleic Acids Res.

[B32] Morozov AV, Siggia ED (2007). Connecting protein structure with predictions of regulatory sites. Proc Natl Acad Sci USA.

[B33] Schultz SC, Shields GC, Steitz TA (1991). Crystal structure of a CAP-DNA complex: the DNA is bent by 90 degrees. Science.

[B34] Maris AE, Sawaya MR, Kaczor-Grzeskowiak M, Jarvis MR, Bearson SM, Kopka ML, Schroder I, Gunsalus RP, Dickerson RE (2002). Dimerization allows DNA target site recognition by the NarL response regulator. Nat Struct Biol.

[B35] Schumacher MA, Choi KY, Zalkin H, Brennan RG (1994). Crystal structure of LacI member, PurR, bound to DNA: minor groove binding by alpha helices. Science.

[B36] Fujikawa N, Kurumizaka H, Nureki O, Terada T, Shirouzu M, Katayama T, Yokoyama S (2003). Structural basis of replication origin recognition by the DnaA protein. Nucleic Acids Res.

[B37] Murzin AG, Brenner SE, Hubbard T, Chothia C (1995). SCOP: a structural classification of proteins database for the investigation of sequences and structures. Journal of molecular biology.

[B38] Mahony S, Benos PV (2007). STAMP: a web tool for exploring DNA-binding motif similarities. Nucleic Acids Res.

[B39] Contreras-Moreira B, Branger PA, Collado-Vides J (2007). TFmodeller: comparative modelling of protein-DNA complexes. Bioinformatics.

[B40] Thieffry D, Salgado H, Huerta AM, Collado-Vides J (1998). Prediction of transcriptional regulatory sites in the complete genome sequence of Escherichia coli K-12. Bioinformatics.

[B41] Sarai A, Kono H (2005). Protein-DNA recognition patterns and predictions. Annu Rev Biophys Biomol Struct.

[B42] Donald JE, Chen WW, Shakhnovich EI (2007). Energetics of protein-DNA interactions. Nucleic Acids Res.

[B43] Miller JC, Pabo CO (2001). Rearrangement of side-chains in a Zif268 mutant highlights the complexities of zinc finger-DNA recognition. Journal of molecular biology.

[B44] Siggers TW, Silkov A, Honig B (2005). Structural alignment of protein–DNA interfaces: insights into the determinants of binding specificity. Journal of molecular biology.

[B45] Lozada-Chavez I, Angarica VE, Collado-Vides J, Contreras-Moreira B (2008). The role of DNA-binding specificity in the evolution of bacterial regulatory networks. Journal of molecular biology.

[B46] McDonald IK, Thornton JM (1994). Satisfying hydrogen bonding potential in proteins. Journal of molecular biology.

[B47] Lu XJ, Olson WK (2003). 3DNA: a software package for the analysis, rebuilding and visualization of three-dimensional nucleic acid structures. Nucleic Acids Res.

[B48] Olson WK, Gorin AA, Lu XJ, Hock LM, Zhurkin VB (1998). DNA sequence-dependent deformability deduced from protein-DNA crystal complexes. Proc Natl Acad Sci USA.

[B49] Gama-Castro S, Jimenez-Jacinto V, Peralta-Gil M, Santos-Zavaleta A, Penaloza-Spinola MI, Contreras-Moreira B, Segura-Salazar J, Muniz-Rascado L, Martinez-Flores I, Salgado H (2008). RegulonDB (version 6.0): gene regulation model of Escherichia coli K-12 beyond transcription, active (experimental) annotated promoters and Textpresso navigation. Nucleic Acids Res.

[B50] van Helden J (2003). Regulatory sequence analysis tools. Nucleic Acids Res.

[B51] Bower MJ, Cohen FE, Dunbrack RL (1997). Prediction of protein side-chain rotamers from a backbone-dependent rotamer library: a new homology modeling tool. Journal of molecular biology.

[B52] Crooks GE, Hon G, Chandonia JM, Brenner SE (2004). WebLogo: a sequence logo generator. Genome Res.

[B53] MacIsaac KD, Wang T, Gordon DB, Gifford DK, Stormo GD, Fraenkel E (2006). An improved map of conserved regulatory sites for Saccharomyces cerevisiae. BMC Bioinformatics.

[B54] Strunk B, Struffi P, Wright K, Pabst B, Thomas J, Qin L, Arnosti DN (2001). Role of CtBP in transcriptional repression by the Drosophila giant protein. Dev Biol.

